# Palmar fracture-dislocation of the trapezoid with median nerve contusion. Case report and literature review

**DOI:** 10.1080/23320885.2021.1927739

**Published:** 2021-05-19

**Authors:** Sara Montanari, Leone Pangallo, Annalisa Valore, Roberto Adani

**Affiliations:** a Department of Hand Surgery and Microsurgery, Azienda Ospedaliero Universitaria Modena, Modena, Italy; b Department of Hand Surgery, Azienda Ospedaliero Universitaria Verona, Verona, Italy

**Keywords:** Complex carpal injuries, palmar trapezoid dislocation, scaphotrapezio-trapezoid dislocation, median nerve, acute carpal tunnel syndrome

## Abstract

Complete dislocations of the trapezoid are very uncommon injuries. The authors present a case of open palmar trapezoid fracture-dislocation with significant displacement of the fracture, acute carpal tunnel syndrome and other concomitant carpometacarpal injuries. A review of the literature search for palmar trapezoid dislocations and treatments was performed.

## Introduction

Trapezoid dislocations are rare injuries and two-third of these are dorsally dislocated [1]. We report a case of complex carpal injury that consists of open palmar fracture-dislocation of trapezoid with fragment migration, multiple carpometacarpal joints dislocation and acute carpal tunnel syndrome. These injuries are extremely rare and are only sporadically described in the literature. This pattern resulted from a severe crush injury. All the cases of palmar dislocation of the trapezoid previously reported in the English literature are reviewed and we did not identify a similar case reported.

## Case report

**Figure 1.**
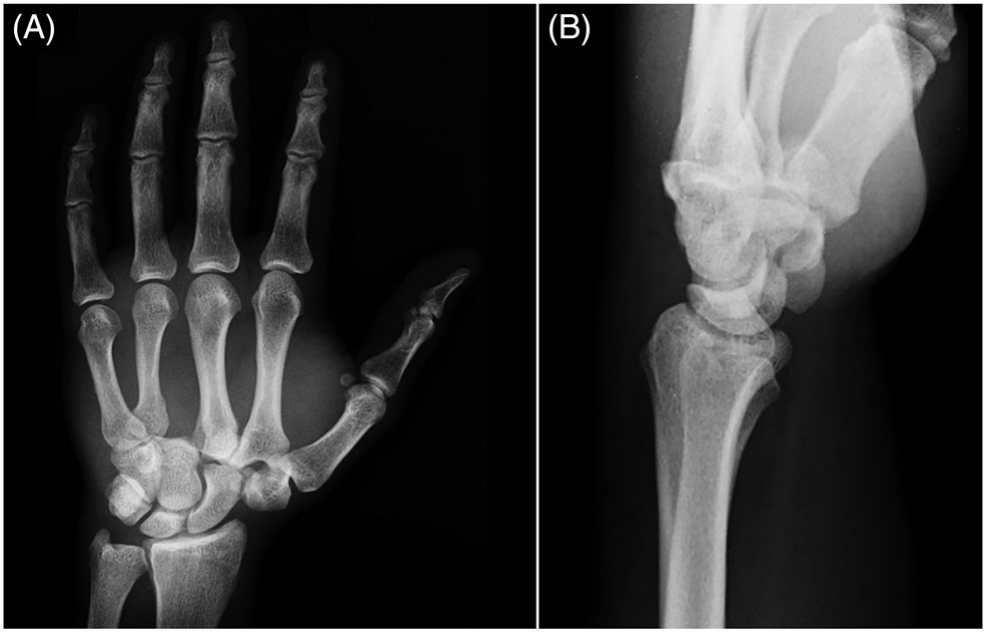
Pre-reduction AP and lateral radiograph of the left wrist.

A 38-year-old male metalworker was presented to our care following a crushing trauma on his left hand in an industrial press machine. He was unable to describe the exact mechanism of the injury. On physical examination, there was severe swelling of the hand and wrist with palpable bony prominence on the dorsal aspect of the carpus, and a wound on the ulnar aspect of the palm, volar to the fourth metacarpal. The patient also referred paresthesias and decreased sensation to light touch in the median nerve distribution of the hand. Routine radiographs revealed dorsal dislocations of the carpometacarpal joints of the index, long and ring fingers with radial migration of the second and third metacarpal bases, scapho-trapezium joint dislocation and fracture-dislocation of the trapezoid (Figure 1). A CT scan was performed to further characterize the injuries. Two days following the accident, the patient underwent surgery under regional brachial plexus block.

Open reduction and median nerve release were performed through a dorsal double incision, combined to a volar one.

The first longitudinal incision was made dorsal to the wrist, centered on third intermetacarpal space (over the 3/4 CMC joint), and was used to reduce and stabilize dislocations of the third and fourth metacarpals with multiple K-wires and dorsal capsulodesis.

**Figure 2.**
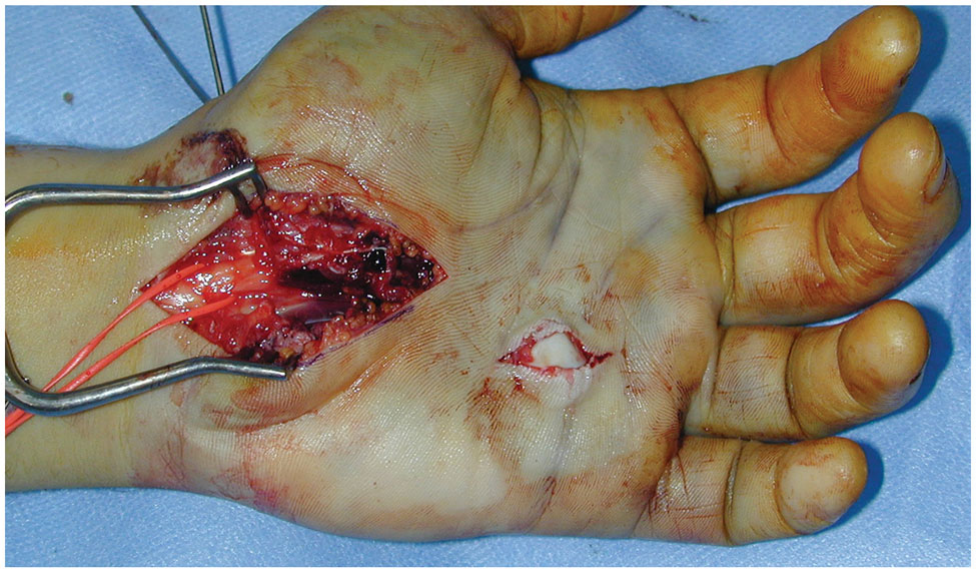
Intraoperative photograph of the migrated trapezoid fragment which caused the cutaneous laceration in the palm. After decompression the median nerve appears hemorrhagic, but in continuity.

Then, an extended carpal tunnel incision was made and open carpal tunnel release and neurolysis of the median nerve were performed. The nerve sustained a blunt contusion from the neighbouring dislocated bones and was suffering due to increasing intracarpal tunnel pressure. The intraoperative features showed focal swelling and hyperemia of the nerve, without crushing or lesion in continuity of the axons. The trapezoid was extruded volarly and split into two fracture fragments; one of which was occupying the carpal tunnel and the other was migrated distally and ulnarly in the palm (Figure 2). The first fragment maintained a connection with the volar ligaments.

**Figure 3.**
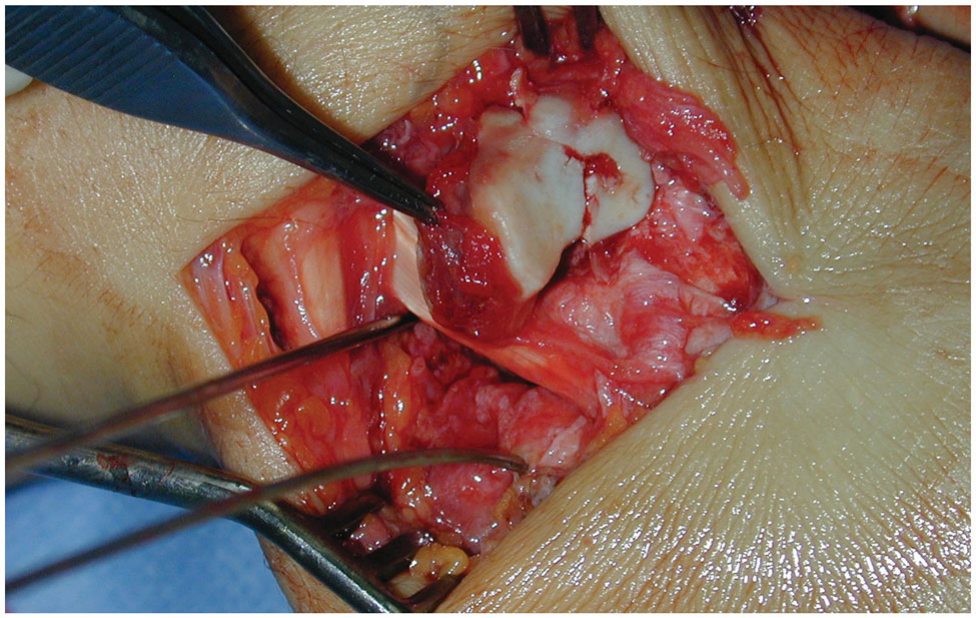
Intraoperative reduction of the trapezoid before screw stabilization.

**Figure 4.**
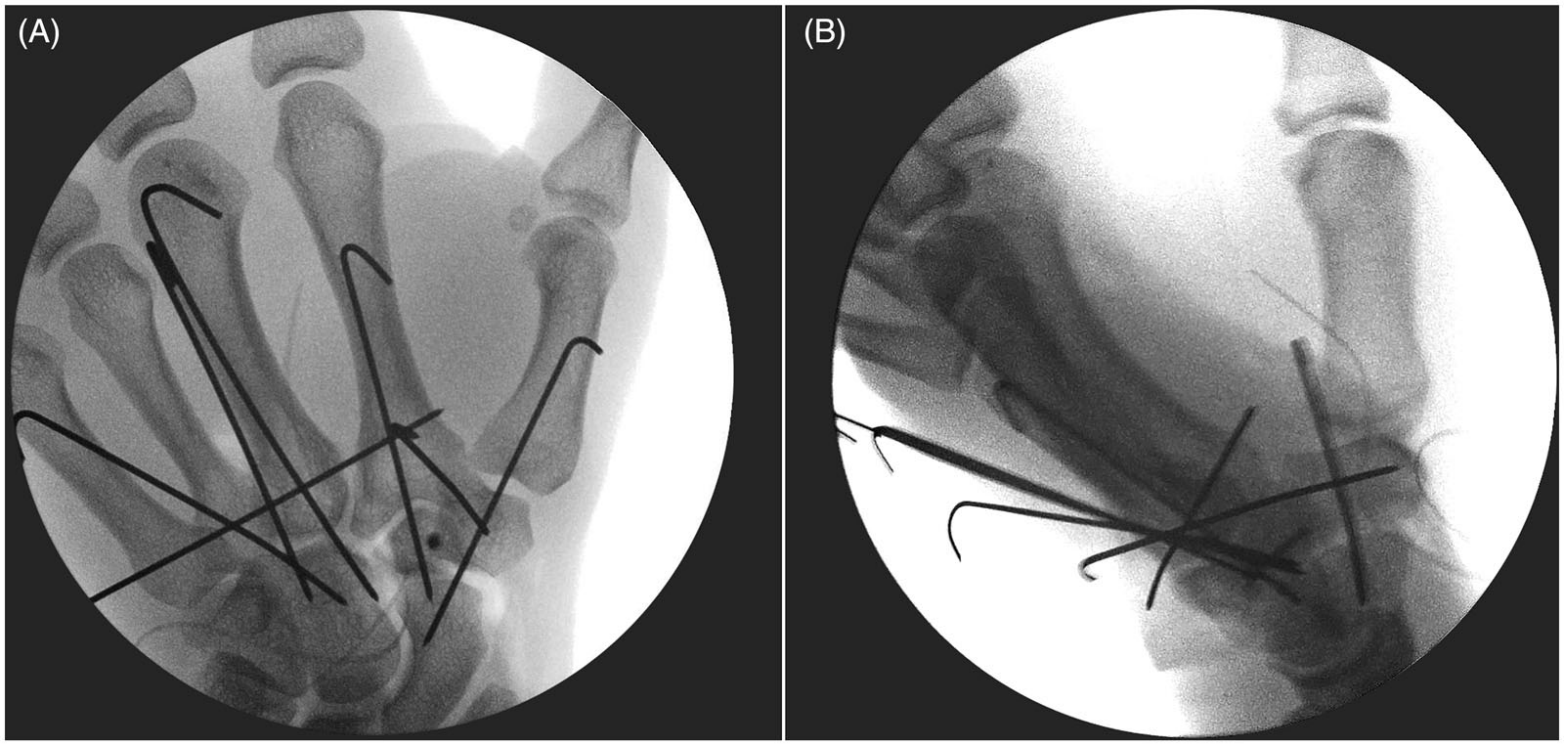
AP and lateral radiographs following fixation, highlighting restoration of the anatomy and full length of the index and long rays.

**Figure 5.**
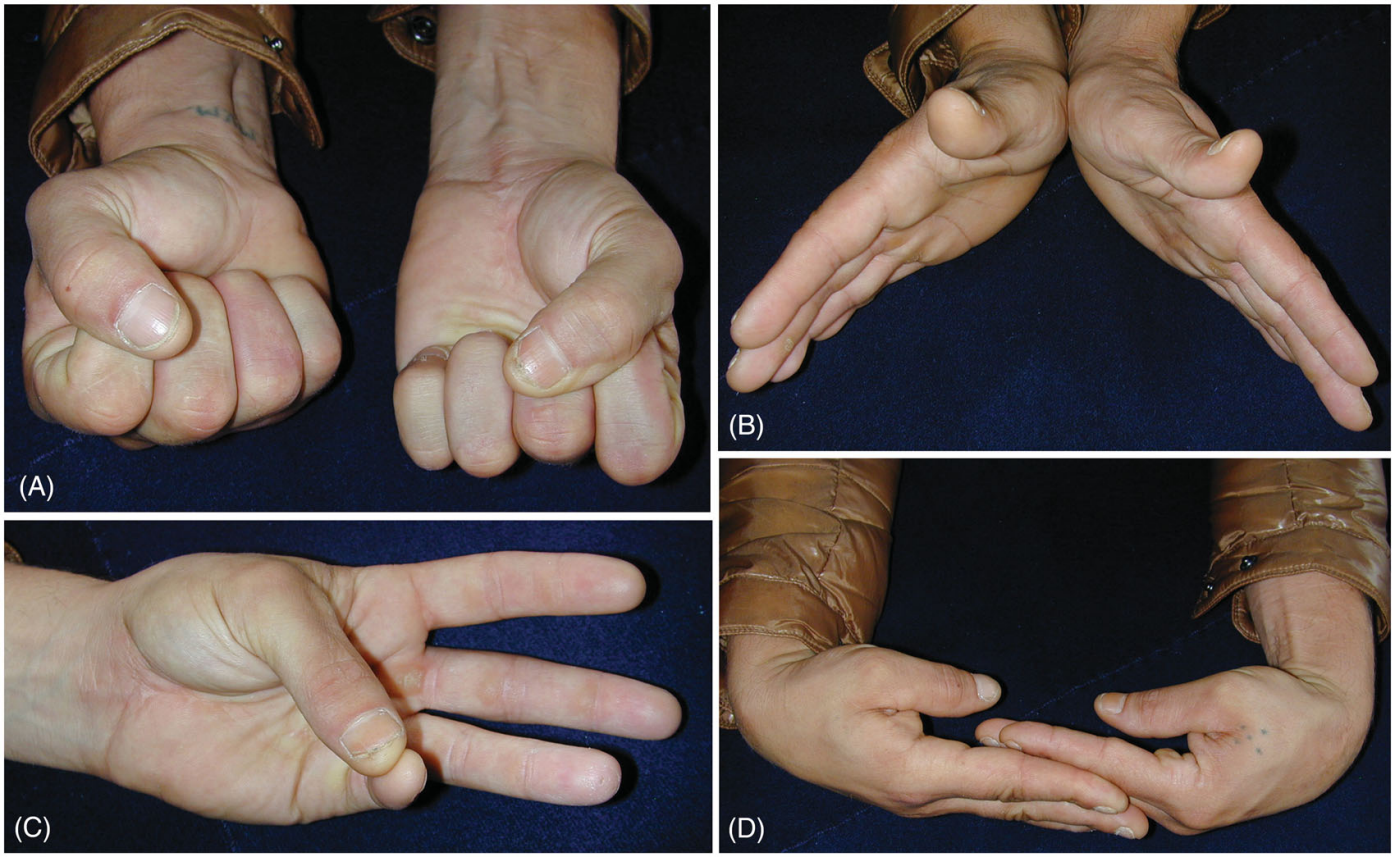
Clinical photographs two years after surgery showing good functional recovery.

**Figure 6.**
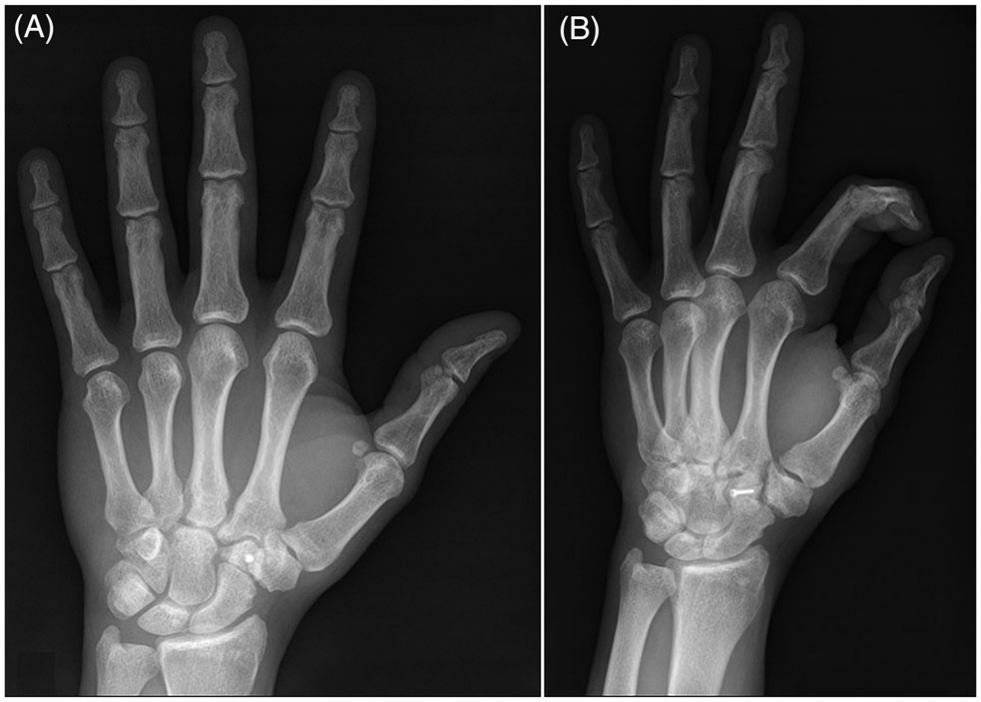
Radiographs of the left hand at the two years follow-up visit demonstrating congruency of all carpal bones and carpometacarpal joints and no signs of avascular necrosis or arthritis.

A second dorsal longitudinal wrist incision was made, centered on first intermetacarpal spaces. Hemi-trapezoid was recovered from the palm, reduced and fixed with a 1.2-mm OsteoMed mini screw (Figure 3). Afterwards, the trapezoid was relocated, trapeziometacarpal and scaphotrapezial joints were reduced and stabilized with K-wires and avulsed dorsal ligaments were repaired. Intraoperative radiographs revealed successful reduction (Figure 4). The wrist was immobilized in a volar splint for four weeks, at which time the pins were removed and an intensive rehabilitation program was initiated. The patient returned to work after four months. Two years following the operation, he has a painless wrist with a good functional outcome. Functionally, the range of motion compared with the uninjured hand was complete in palmar flexion, radial and ulnar deviation, pronation and supination. The exception was noted with the dorsiflexion where 50° was achieved compared to 70° in the uninjured hand. Opposition of the thumb to the small finger was good; a Kapandji score of 8 was registered (Figure 5). The Disabilities of the Arm, Shoulder and Hand (DASH) score was 1.7 and DASH work module was 0.0. Grip strength of both extremities, as measured by the Jamar dynamometry (level 3) and Pinch tests, resulted in an average of 43 kg vs 46.6 kg and 11 kg vs 9 kg for the injured and uninjured hands, respectively. The patient had no signs of any median nerve dysfunction and normal 2-p discrimination in the median nerve distribution. Radiographs at two-years follow-up showed good alignment and no evidence of avascular necrosis or arthritis (Figure 6). This study is performed in accordance with the Ethical Standards of the 1964 Declaration of Helsinki. The patient gave written, informed consent to report data from this case.

## Discussion

Complete palmar dislocation of the trapezoid is a rare injury resulting from high-energy trauma. Our review of the English literature in PubMed database found twelve reports on this topic, all reported in Table 1 [1–12]. Only two of these patients had an isolated injury [6,9]. The other reports had associated injuries, including several cases of metacarpal fractures or carpometacarpal dislocations, some cases of intercarpal dislocations, one case of open dislocation [4], one case of Galeazzi fracture [10], one case of attritional rupture of the flexor tendons to the index finger [8] and one case of acute carpal tunnel syndrome [1]. Recent literature has attempted to provide improved description of the traumatic mechanism, but it is still unclear. The trapezoid is a wedge-shaped bone whose dorsal surface area is about twice of its volar surface area. It is in a well-protected position between the trapezium, scaphoid, capitate, and index metacarpal and has strong ligament attachments that bind it to the adjacent bones. The volar intercarpal ligaments are the strongest. Consequently, injuries to the trapezoid are rarely seen and there is no clear explanation as to how a wedge-shaped bone wider dorsally dislocates palmarly. In fact, two-third of these injuries are dorsal dislocations [1,11]. The mechanism postulated for dorsal dislocations is a force applied to the distal dorsal end of the second metacarpal with the wrist in slight flexion. This force acts as a lever and displaces the trapezoid, allowing the proximal migration of second metacarpal toward the scaphoid and the scaphotrapezial joint. The trapezium and the trapeziometacarpal complex may dislocate radially and proximally. Disruption of the scaphotrapezial joint as proposed by Laing et al. [13] could represent the extension of the trapezoid dislocation mechanism. Palmar dislocation of the trapezoid has been postulated to occur by means of a direct blow on the dorsal trapezoid causing flattening of the carpal arch and extrusion of the trapezoid or by forced hyperextension of the midcarpal joint [14]. To our knowledge, the current case represents the first description of fracture and complete palmar dislocation of the trapezoid with acute carpal tunnel syndrome. The singularity of a significant displacement of the fracture and the association with multiple carpometacarpal dislocations and scapho-trapezium joint dislocation are also present. In this case, preoperative radiographs obtained in the emergency department including a posteroanterior view of the hand and a lateral view of the wrist (Figure 1) provided an incomplete diagnosis. We were not able to visualize the correct location of trapezoid on the standard preoperative views because of overlap. Even in case of a slight displacement, the bone superposition of carpal bones and the common presence of several associated injuries make CT scan often necessary and generally recommended [15].

**Table 1.** Published Data on Complete Palmar Dislocation of the Trapezoid.

*n*	Year	Author	Age	Associated injuries	Treatment	Follow-up (mo)	Recovery	Complications
**1**	1962	Lewis [2]	51	Open fracture of the IV MC, dislocation of the II and III CMC joints	Excision of the trapezoid, pinning	36	Pain and weakness	Proximal migration of the index MC
**2**	1983	Rhoades and Reckling [3]	67	Fractures of the I, IV, V MC, dislocation of the II CMC joint	Open reduction, Pinning Ligament repair	12	Pain, good ROM - opposition, weak grip strength	Avascular necrosis
**3**	1983	Goodman and Shankman [4]	37	Open palmar trapezoid dislocation, dorsal dislocation of the II and III MC	Open reduction, limited wrist arthrodesis	12	Intermittent pain, limited ROM, weak grip strength	*
**4**	1985	Dunkerton and Singer [5]	23	Dorsal dislocation of the II MC	Open reduction	2	No pain, good ROM	Sclerotic changes (avascular necrosis?)
**5**	1985	Kopp [6]	22	/	Open reduction, Ligament repair	6	No pain, good ROM, normal grip strength	*
**6**	1989	Yao and Lee [7]	21	Radial subluxation of the trapezium	Open reduction, Pinning	48	No pain or functional deficit	Subluxation and degenerative changes at the II CMC joint
**7**	1990	Inoue and Inagaki [8]	57	Attritional rupture of flexor tendons to the index finger	Excision of the trapezoid	144	No pain, limited ROM, weak grip strength	Proximal migration of the index MC, degenerative changes in the mid-carpal joint.
**8**	1992	De Tullio and Celenza [9]	21	Trapezoid fracture-dislocation	Open reduction, Pinning	4	Full function	*
**9**	1998	Taylor and Shakespeare [10]	28	Galeazzi fracture-dislocation, fractures of the IV and V MC, fracture of distal capitate pole, mild median nerve paresthesia	Open reduction, Pinning, Ligament repair	3	No pain, limited ROM, normal 2-p discrimination	Avascular necrosis?
**10**	2003	Koenig and West [11]	39	II, III, IV MC subluxation, fracture of the triquetrum	Open reduction, Pinning Ligament repair	lost	/	Subluxation of the trapezoid, II, III and IV CMC joints
**11**	2005	Larson and DeLange [1]	21	Acute carpal tunnel syndrome	Open reduction, pinning	6	No pain, good ROM and grip strength, normal 2-p discrimination	*
**12**	2008	Calfee et al [12]	45	Dorsal trapezoid fracture, scaphoid's distal pole, hook of hamate fracture, mild median nerve paresthesia	Open reduction, mini-screw fixation, Ligament repair	7	Slight limitation in ROM and grip strength	*
**13**	2020	Present study	38	Open palmar trapezoid fracture-dislocation, dislocation of the scapho-trapezium, II, III and IV CMC joints, acute carpal tunnel syndrome	Open reduction, Pinning Ligament repair	24	No pain, good ROM, normal grip strength, normal 2-p discrimination	*

MC: metacarpal; CMC: carpometacarpal; ROM: range of motion

*indicates absence of the condition in the original study.

Table 1 denotes that treatments used for volar dislocations have included open reduction and K-wires fixation with or without ligament repair [1,3,7,9–11], trapezoid excision [2,8], and limited wrist arthrodesis [4]. Excision of the trapezoid is contraindicated because of proximal migration of the index metacarpal into the empty space of the trapezoid and establishment of wrist's instability and degenerative changes [2,8]. Limited wrist fusion (trapezoid-trapezium-capitate-index and middle metacarpal bases with a cancellous bone graft taken from the radial styloid) has been used by Goodman and Shankman [4] exploiting the limited joint mobility of this area. We believe that this procedure should be considered to be a salvage procedure and should be reserved for patients with complications, such as symptomatic degenerative arthritis. When dislocated palmarly, closed reduction of the trapezoid has never been successful because of the shape of the bone [2–4,6–9,11,12]. Open reduction through a dorsal approach is always necessary. Despite the differences in injury patterns, description of the results and follow-up periods between the studies we believe that open reduction associated with multiple K-wires stabilization and dorsal capsulodesis generally achieves good functional results. The outcomes are generally satisfactory, but diminished range of motion of the wrist and thumb, as well as decreased grip and key pinch strength, should be preoperatively anticipated. Complications include residual instability, loss of reduction, mid-carpal degenerative changes and avascular necrosis of the trapezoid. In case of fracture, delayed union or nonunion of the trapezoid and posttraumatic arthritis are also possible.
